# The potential role of central obesity in male infertility: body mass index versus waist to hip ratio as they relate to selected semen parameters

**DOI:** 10.1186/s12889-020-8413-6

**Published:** 2020-03-12

**Authors:** Márton Keszthelyi, V. Anna Gyarmathy, András Kaposi, Zsolt Kopa

**Affiliations:** 1 Department of Urology, Andrology Centre, Semmelweis University, Üllői út 78/b, Budapest, 1082 Hungary; 2 EpiConsult LLC, 8 The Green, STE A, Dover, DE 19904 USA; 3 Johns Hopkins Bloomberg School of Public Health, Baltimore, 615 N Wolfe St, Baltimore, MD 21205 USA; 4 Department of Biophysics and Radiation Biology, Semmelweis University, Tűzoltó u. 37-47, Budapest, 1094 Hungary; 5 Department of Urology, Andrology Centre, Semmelweis University, Üllői út 78/b, Budapest, 1082 Hungary

**Keywords:** Male infertility, Statistical modelling, Obesity, Body mass index, Waist to hip ratio

## Abstract

**Background:**

Little is known about the potential role of central obesity among men. Our first aim was to confirm what is already known from prior research, namely that both BMI and WHR are inversely associated with selected semen parameters. Our second aim was to examine the potential role of central obesity by assessing if there was a difference between BMI and WHR regarding their relationships to these selected semen parameters.

**Methods:**

In this cross-sectional study between January 2011 to January 2018, we analyzed semen samples from 1169 patients who visited an andrology clinic in Budapest for infertility reasons. Variables assessed were: body measurements (height, weight, waist circumference, and hip circumference), and the results of semen analysis (sperm concentration, total sperm count, progressive sperm motility, and normal sperm morphology).

**Results:**

The mean height and weight were 180.6 cm and 87.3 kg, respectively – the mean BMI was 26.8. The mean waist and hip circumferences were 100.9 cm and 94.8 cm, respectively – the mean waist to hip ratio was 0.94. The mean sperm concentration, total sperm count, and percents of progressive motility and normal morphology were 48.7 M/ml, 165 million, 21.2, and 4.8%, respectively. Both BMI and WHR were significant correlates in all semen parameter regression models. When comparing the parameter estimates for BMI with those for WHR for each semen parameter, the parameter estimate for WHR was significantly lower (indicating a stronger negative association) than that for BMI for progressive motility and total sperm count, but not for normal morphology or concentration.

**Conclusions:**

Our study is the first to examine, using a large patient sample, the potential role of central obesity by comparing the difference between BMI and WHR as they relate to selected semen parameters. Our findings indicate a potential role of central obesity for progressive motility and total sperm count, but not for normal morphology and concentration. Despite the limitations and the exploratory nature of this study, we can conclude that our results point to a potential role of central obesity in male infertility, but this finding should be confirmed and further explored in future research.

**Trial registration:**

The trial was retrospectively authorized after the data collection on September 24, 2018. Registration number: SE RKEB: 169/2018.

## Background

An infertile relationship is defined by the World Health Organization (WHO) as the inability of a couple to achieve spontaneous pregnancy in 1 year despite being sexually active and non-contracepting [1]. Infertility is a serious health problem: in western developed countries about 15% of couples seek medical treatment because of infertility [2]. Several factors are known to be associated with both female and male fertility, including increased body weight [3–5]. For example, men who are overweight are more likely to have abnormal sperm characteristics, such as impairments in – among others – sperm concentration, progressive motility, and normal morphology [6–14].

There are various ways to measure obesity. Body Mass Index (BMI), which is a person’s weight in kilograms divided by the square of their height in meters, is the most frequently used marker [15]. The BMI, however, does not take into consideration certain factors, such as fat distribution and central obesity, which not only mark more precisely the overweight status, but have also been associated with health impairment [16]. The waist to hip ratio (WHR), which is the waist circumference divided by the hip circumference, however, takes into account the differences in body structure and has a proven to have more sensitivity in the prediction of several disease mortalities [17, 18].

Most studies that assessed the relationship between fertility and excess weight have relied on the BMI as a measure for overweight [19], and therefore little is known about the potential role of central obesity, which is better reflected by the WHR. Our study is designed to fill that gap in knowledge. Our first aim was to confirm what is already known from prior research, namely that both BMI and WHR are inversely associated with selected semen parameters. We chose sperm concentration, progressive motility, and normal morphology as semen parameters of interest because these sperm parameters indicate well the status of the sperm, from which we can assess fertility. Our second aim was to examine the potential role of central obesity by assessing if there was a difference between BMI and WHR regarding their relationships to these selected semen parameters. In other words, when BMI and WHR are standardized in order to become comparable with each other, any significant difference, favoring WHR, in the statistical parameters of their respective associations with any given semen parameter would indicate a role of central obesity.

## Methods

Between January 2011 and January 2018 altogether 1188 patients visited an andrology clinic in Budapest, Hungary, for infertility reasons, providing altogether 1345 semen samples. Our center is a certified training center of the European Academy of Andrology and continuously takes part in various international quality control efforts. QuaDeGa and Gamete Expert Andrology Scheme are the running quality controls at our department.

This analysis used chart review of all these patients, which included the following variables: date of visit, date of birth, body measurements (height, weight, waist circumference, and hip circumference), and the results of semen analysis (sperm concentration, total sperm count, progressive sperm motility, and normal sperm morphology) according to current WHO criteria. Age was calculated by subtracting the date of birth from the date of visit.

### Body measurements

Height was measured in centimeters with a standardized cloth tape measure. Patients were asked to remove shoes and stand erect with their shoulders relaxed and looking straight ahead. Weight was measured in kilograms using standardized digital scales. BMI was then calculated by dividing the weight in kilograms with the square of the height in meters (kg/m2). We measured the hip and waist circumference in centimeters according to the WHO 2011 guidelines by means of a constant 100 g tension providing tape [20]. Waist circumference was measured at the midpoint between the lower margin of the last palpable rib and the top of the iliac crest. Hip circumference was measured around the widest portion of the buttocks, with the tape parallel to the floor. WHR was then calculated by dividing the waist circumference measurement with the hip measurement (W ÷ H).

### Semen analysis

Semen analysis was performed according to the WHO Laboratory Manual for the Examination and Processing of Human Semen 5th edition (2010) [1]. The “Who Laboratory manual for the Examination and processing of human semen” 2010 edition declares that to achieve best results for a standard semen analysis, the sample should be collected after a minimum of 2 days and a maximum of 7 days of sexual abstinence [1]. All our patients adhered our prescribed 3–5 days of abstinence.

From the standardized assessment sperm concentration, progressive sperm motility and normal sperm morphology were selected for further analysis. Sperm concentration was measured in million/milliliters (M/mL) by hemocytometer technique with Neubauer improved cell counting chamber. Diff-Quik® stains were used to evaluate sperm progressive motility and normal morphology. The samples were assessed with 400x magnification on an Olympus CX21 microscope, and progressive motility and normal morphology are expressed as percentage of total cells. Computer-assisted sperm analysis (CASA Sperm Class Analyzer - Microptic Automatic Diagnostic System - Spain) was used on a Nikon Eclipse E200 microscope for the quality control of the data.

### Data management and statistical analysis

The data were quality controlled for repeat visits, data entry errors, and influential outliers. Of the 1345 observations, 157 were removed because they were repeat visits, leaving a total of 1188 observations (equaling the first visits of all patients). Of these, one observation was removed because of missing values, and 18 were removed because of data entry errors. Then, loess local regression smooth curve fit plots were created with the proc. loess procedure in SAS V9.4. to visualize influential outliers. Based on this analysis, two observations were removed because they were influential outliers (for both patients, BMI = 54 and WHR = 1.0 with respective large waist and hip circumferences). This analysis also confirmed that the relationships between semen parameters and BMI/WHR are linear. We further assessed our data in order to remove patients with clinical varicocele, orchiditis, epididymitis, and vesiculitis, but the final cleaned dataset did not contain any patients with these conditions. Therefore, the final study sample comprised of 1169 patient observations (98.4% of all patients).

Patient semen parameters were compared by degree of obesity in the following groups: normal weight with BMI less than 25 (438 patients), overweight with BMI between 25 and 29.9 (510 patients) and obese with a BMI above 30 (221 patients), and WHR < = 0.9 (361 patients) and WHR > 0.9. Differences were evaluated with the Kruskal-Wallis non-parametric test.

To compare the regression slopes of the BMI vs. WHR models for each semen parameter, we standardized the BMI and WHR values to range from 0 to 1 using the proc. stdize procedure in SAS V9.4 with the method = range option. We chose this standardization method, because this strictly follows the original distribution of the original variable and makes the two different variables comparable. That means, if we plotted any parameter against either BMI or WHR, the plot with the original values would be identical to the plot with the standardized values, except for the value labels on the axis for BMI/WHR. As such, we included two x axes with our figures: one with the standardized values and one with the original values.

Univariate contingency tables to describe distribution and means procedures were conducted. After the removal of the influential outliers (as described above), the relationship between BMI and WHR, and semen parameters could be fitted as linear. Therefore, scatter plots were created with fitted linear regression lines in order to visualize the relationship between BMI and WHR, and semen parameters. Univariate liner regression models adjusted for age (which is strongly associated both with BMI and WHR, and most likely with indicators for infertility as well) were created to calculate the regression line slope coefficients and their 95% confidence intervals. For reference purposes, we are showing both non-standardized and standardized values for parameter estimates and their confidence intervals, standard errors, t values, *p* values. For each semen parameter (dependent variable), min/max line plots were created comparing the slope coefficients of BMI vs. WHR (independent variables) for that particular parameter. Statistically significant (*p* < 0.05) differences between the slopes were considered when the point estimate of the WHR slope coefficient fell outside of the 95% confidence interval of the BMI slope coefficient [21]. SAS V9.4 software (SAS Institute Inc. Cary, NC) was used for data management and analysis, and data visualization.

## Results

### Description of the sample

Sample characteristics are presented in the Table 1. with means, SDs and data ranges. The mean age of the 1169 patients was 38.1 years. The mean height and weight were 180.6 cm and 87.3 kg, respectively – the mean BMI was 26.8. The mean waist and hip circumference were 100.9 cm and 94.8 cm, respectively – the mean waist to hip ratio was 0.94. The mean sperm concentration and total sperm count were 48.7 M/ml and 164.9 M, respectively. The mean percent of progressive motility and normal morphology were 21.2% and 4.8, respectively.

**Table 1:** Characteristics of the sample (*N* = 1169)

Characteristic	mean (SD)	Range
Participants		
Age	38.1 (7)	17–67
Height	180.6 (7.5)	155–210
Weight	87.3 (15.9)	55–183
BMI	26.8 (4.5)	16.9–50
Waist circumference	100.9 (8.9)	56–149
Hip circumference	94.8 (12.4)	59–165
Waist-to-hip ratio	0.94 (0.07)	0.63–1.22
Specimen		
Semen volume	3.6 (1.8)	0.1–12.0
Total sperm count	165.5 (192.2)	0–1270
Sperm concentration	48.7 (55.7)	0–681.5
Progressive motility	21.2 (18.7)	0–80
Normal morphology	4.8 (4.6)	0–28
WBC	0.59 (5.8)	0–193.1

Table 2 shows mean, SD and range of sperm parameters by BMI and WHR categories and their significance testing. The figures show a decrease of semen parameter values with an increase of BMI and WHR categories.

**Table 2:** Semen parameters distributed by the degree of obesity

	Normal weight BMI < 25 (*N* = 438)		overweight 25 < BMI < 29.9 (*N* = 510)		obese BMI > 30 (*N* = 221)		Kruskal-Wallis
	Mean (SD)	Range	Mean (SD)	Range	Mean (SD)	Range	*p*-value
Sperm concentration (M/ml)	49.6 (50.14)	0–295.25	51.2 (63.5)	0–681.5	41.1(45.7)	0–256	0.0362
Total sperm count (M)	174.6 (190.1)	0–1014.3	167.3 (205.4)	0–1270	140.2(160.6)	0–752.6	0.0239
Progressive motility (%)	22.3 (18)	0–77.5	20.9 (19.4)	0–80	19.5 (18.1)	0–75	0.0582
Normal morphology (%)	4.9 (4.2)	0–21.5	4.9 (4.9)	0–28	4.3(4.3)	0–19.5	0.0455
	WHR = < 0.9 (*N* = 361)		WHR > 0.9 (*N* = 808)		Kruskal-Wallis		
	Mean (SD)	Range	Mean (SD)	Range	*p*-value		
Sperm concentration (M/ml)	56.3 (56.9)	0–340	45.3 (54.9)	0–681.5	0.0001		
Total sperm count (M)	201.1 (216)	0–1087.2	148.75(178.3)	0–1270	<.0001		
Progressive motility (%)	24.2(19.1)	0–80	19.9(18.3)	0–80	0.0002		
Normal morphology (%)	5.3(4.9)	0–24	4.6(4.4)	0–28	0.0672		

### Results of regression procedures

**Fig. 1:**
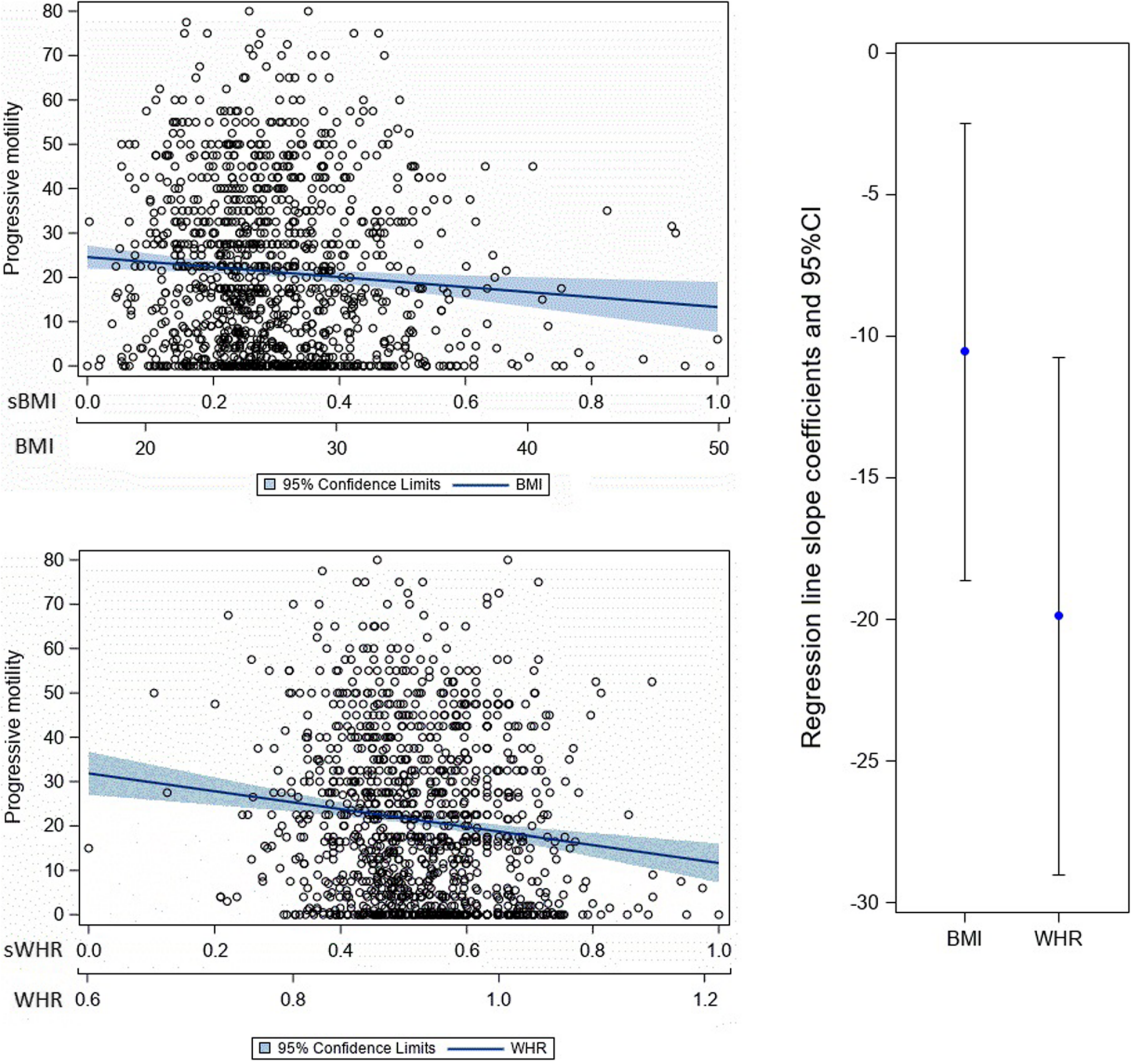
The relationship of progressive motility to body mass index and waist to hip ratio. Note: The top left charts show scatterplots with age-adjusted linear regression lines and their corresponding 95% confidence interval band depicting the association between progressive motility and BMI (top left) and progressive motility and WHR (bottom left). The chart on the right shows the age-adjusted regression line slope coefficients with their 95% confidence intervals contrasting BMI with WHR as they relate to progressive motility

**Fig. 2:**
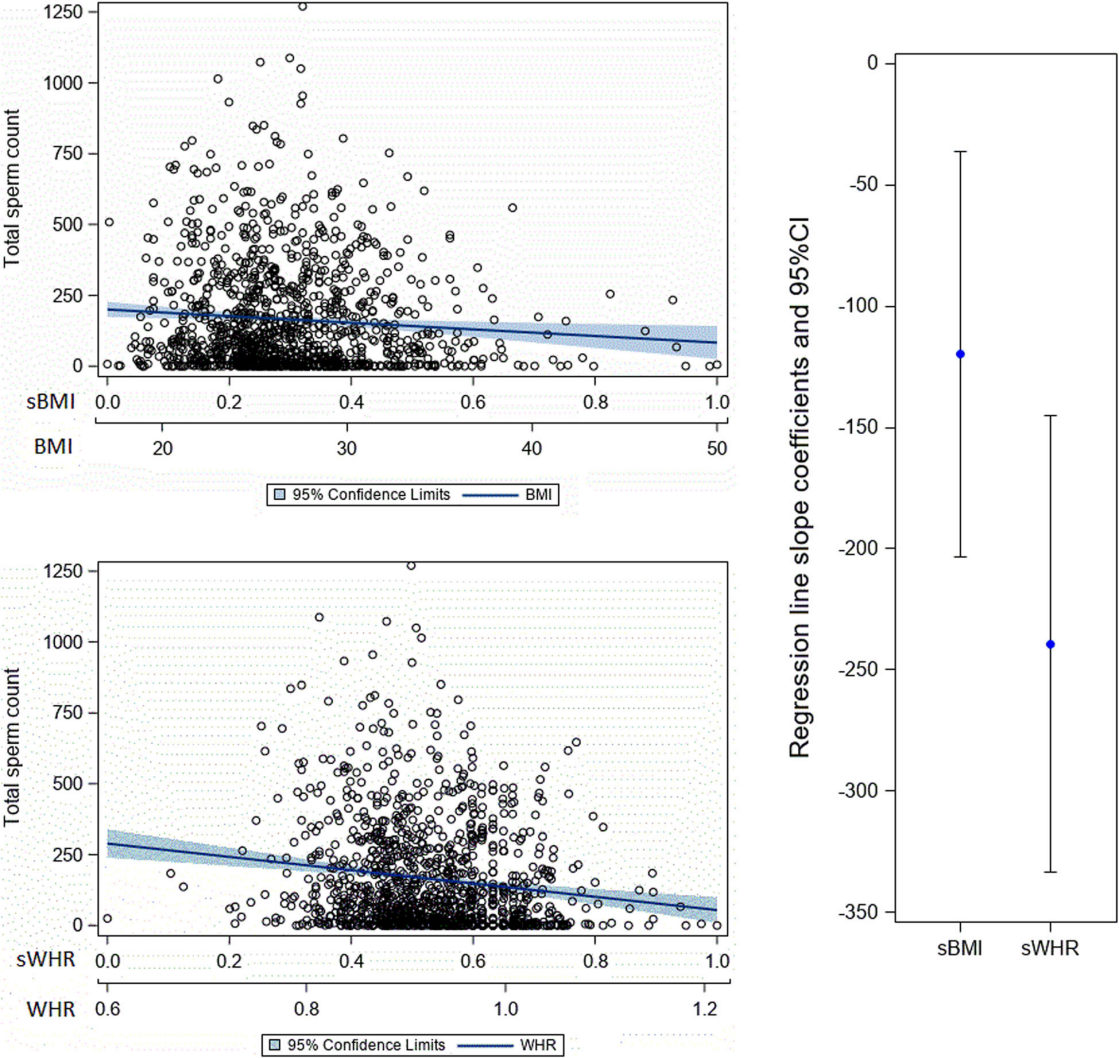
The relationship of total sperm count to body mass index and waist to hip ratio. Note: The top left charts show scatterplots with age-adjusted linear regression lines and their corresponding 95% confidence interval band depicting the association between total sperm count and BMI (top left) and total sperm count and WHR (bottom left). The chart on the right shows the age-adjusted regression line slope coefficients with their 95% confidence intervals contrasting BMI with WHR as they relate to total sperm count

**Fig. 3:**
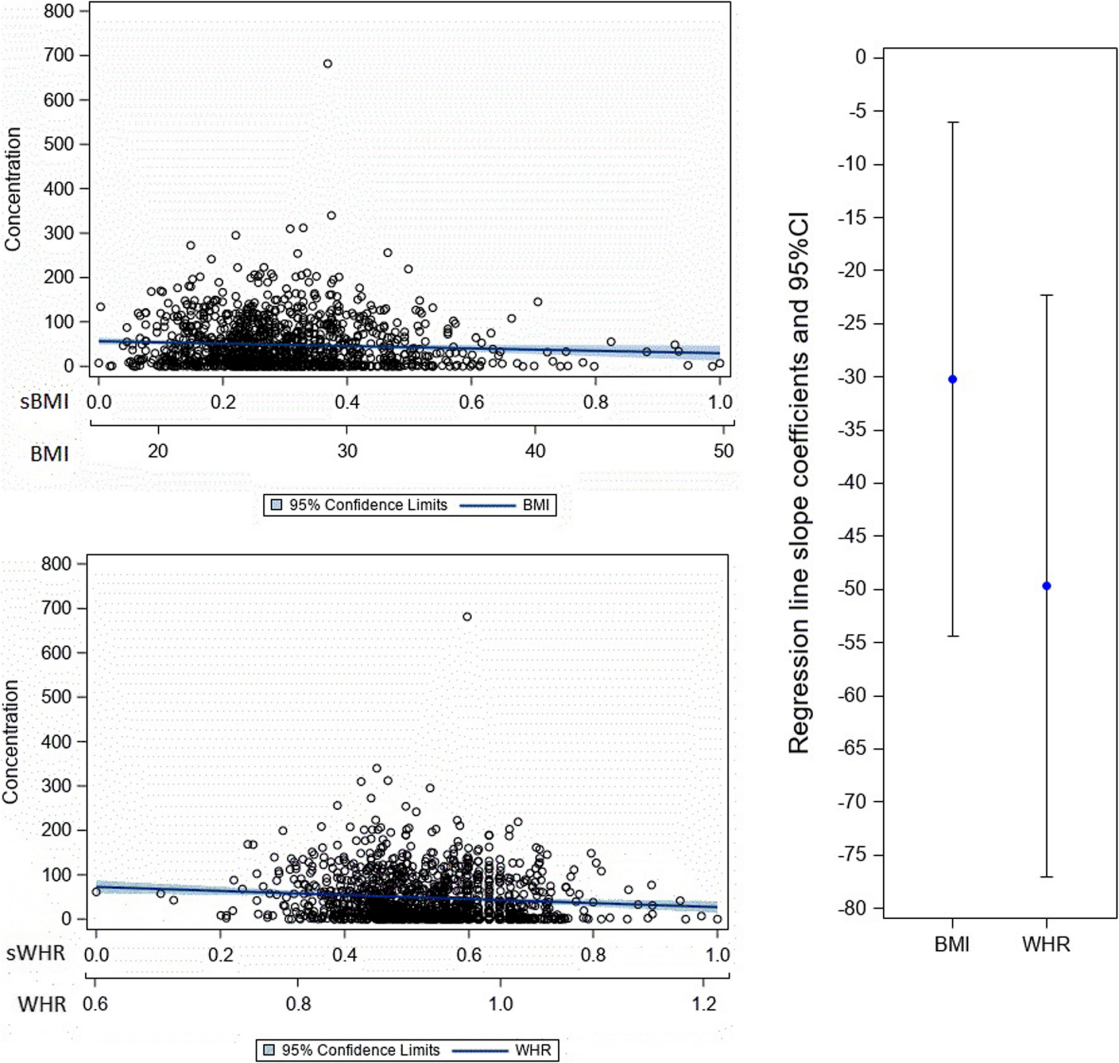
The relationship of concentration to body mass index and waist to hip ratio. Note: The top left charts show scatterplots with age-adjusted linear regression lines and their corresponding 95% confidence interval band depicting the association between concentration and BMI (top left) and concentration and WHR (bottom left). The chart on the right shows the age-adjusted regression line slope coefficients with their 95% confidence intervals contrasting BMI with WHR as they relate to concentration

**Fig. 4:**
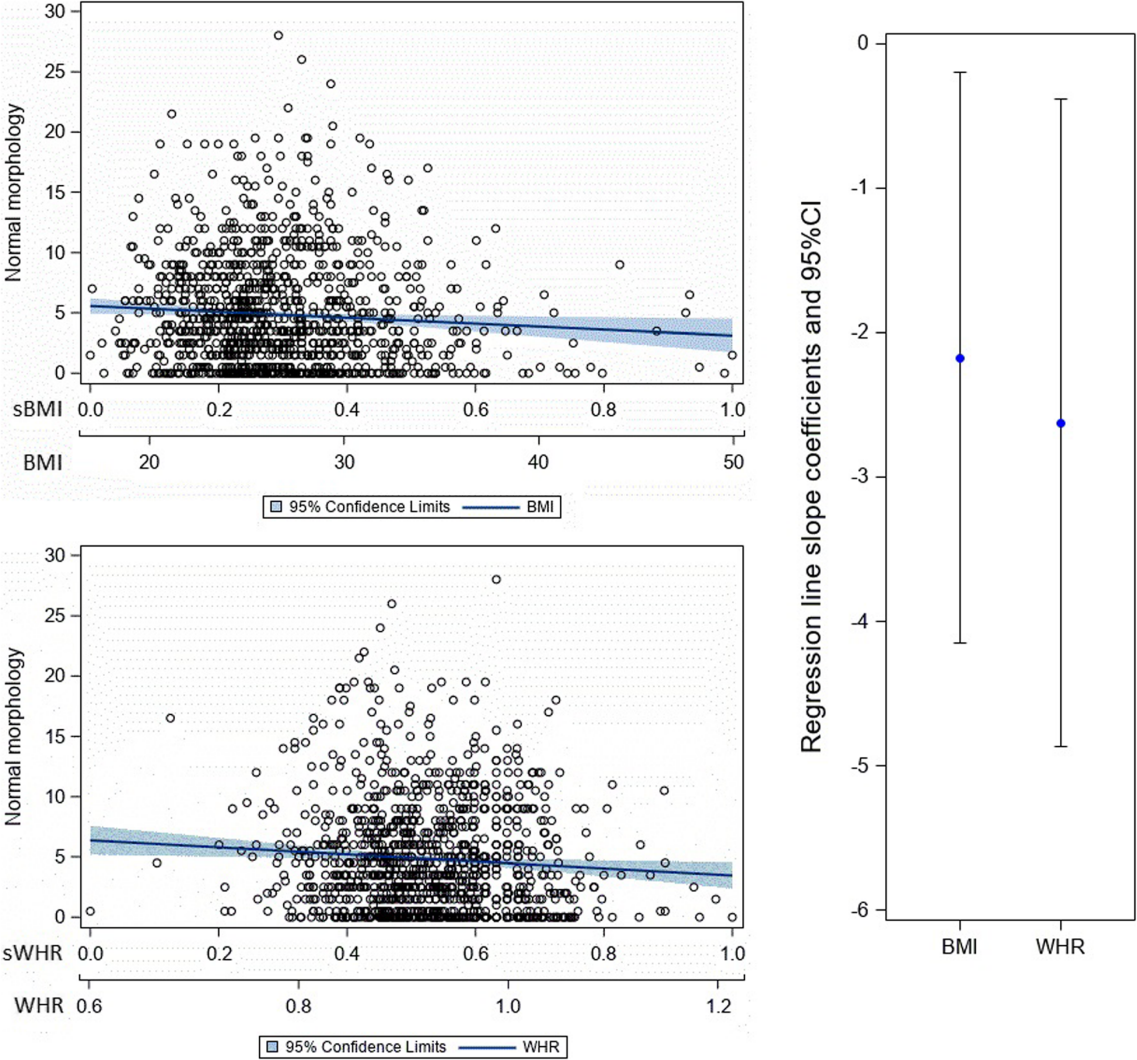
The relationship of normal morphology to body mass index and waist to hip ratio. Note: The top left charts show scatterplots with age-adjusted linear regression lines and their corresponding 95% confidence interval band depicting the association between normal morphology and BMI (top left) and normal morphology and WHR (bottom left). The chart on the right shows the age-adjusted regression line slope coefficients with their 95% confidence intervals contrasting BMI with WHR as they relate to normal morphology

Both BMI and WHR were significant correlates in all age-adjusted semen parameter linear regression models (Table 3). Even though we adjusted for age, it was not significant in the context of concentration and progressive motility, only in the context of normal morphology (meaning higher age was associated with lower morphology values). When comparing the standardized parameter estimates for BMI with those for WHR for each semen parameter, the parameter estimate for WHR was significantly lower (indicating a stronger negative association) than that for BMI for progressive motility and total sperm count (Figs. 1 and 2), but not for concentration (Fig. 3 ) or normal morphology (Fig. 4).

**Table 3:** Linear regression results for both non-standradized and standarzied BMI and WHR values

Non-standarzied Variable	Parameter	95% Confidence Limits		Standard	t Value	Pr > |t|
	Estimate			Error		
Concentration						
BMI	−0.91	−1.65	−0.18	0.37	−2.45	0.01
age	0.19	−0.27	0.66	0.24	0.82	0.41
WHR	−84.93	− 131.71	−38.15	23.84	−3.56	0.00
age	0.24	−0.23	0.70	0.24	1.00	0.32
Progressive motility						
BMI	−0.32	−0.56	− 0.07	0.12	−2.56	0.01
age	−0.11	−0.27	0.04	0.08	−1.43	0.15
WHR	− 33.99	−49.59	− 18.40	7.95	−4.28	<.0001
age	−0.09	− 0.25	0.06	0.08	−1.15	0.25
Normal morphology						
BMI	−0.07	−0.13	− 0.01	0.03	−2.15	0.03
age	−0.04	−0.08	0.00	0.02	−2.09	0.04
WHR	−4.49	−8.32	−0.65	1.95	−2.30	0.02
age	−0.04	−0.08	0.00	0.02	−2.09	0.04
Total sperm count						
BMI	−3.62	−6.14	−1.09	1.29	−2.81	0.01
age	0.05	−1.55	1.66	0.82	0.07	0.95
WHR	− 409.00	− 569.64	− 248.36	81.87	−5.00	<.0001
age	0.36	−1.24	1.95	0.81	0.44	0.66
Standardized Variable	Parameter	95% Confidence Limits		Standard	t Value	Pr > |t|
	Estimate			Error		
Concentration						
sBMI	−30.25	−54.45	−6.05	12.34	−2.45	0.01
age	0.19	−0.27	0.66	0.24	0.82	0.41
sWHR	−49.69	−77.05	−22.32	13.95	−3.56	0.00
age	0.24	−0.23	0.70	0.24	1.00	0.32
Progressive motility						
sBMI	−10.55	−18.63	−2.46	4.12	−2.56	0.01
age	−0.11	−0.27	0.04	0.08	−1.43	0.15
sWHR	−19.89	−29.01	−10.77	4.65	−4.28	<.0001
age	−0.09	−0.25	0.06	0.08	−1.15	0.25
Normal morphology						
sBMI	−2.17	−4.15	−0.19	1.01	−2.15	0.03
age	−0.04	−0.08	0.00	0.02	−2.09	0.04
sWHR	−2.63	−4.87	−0.38	1.14	−2.30	0.02
age	−0.04	−0.08	0.00	0.02	−2.09	0.04

## Discussion

This is the first study to examine, using a large patient sample, the potential role of central obesity by assessing the difference between BMI and WHR as they relate to selected semen parameters. Our study confirmed what has already been known from prior research: both BMI and WHR were inversely associated with our selected semen parameters. Furthermore, our finding that WHR had a significantly stronger negative association with progressive motility than BMI while there was no such difference for normal morphology and concentration indicates a potential role of central obesity for progressive motility but not for the latter two semen parameters.

While the role of central obesity in the decreased fertility rates of women has been well researched [22], little is known about the potential role of central obesity among men. As can be seen in the literature, both BMI and WHR are associated with some markers of infertility but not with others, and BMI and WHR are associated to different degrees with those parameters. For example, although the LIFE study assessed only waist circumference but not WHR, the results show a linear decline of ejaculate volume with increasing BMI and waist circumference, and it appears that the magnitude of the association is similar [23]. They further found that median sperm count was significantly associated with waist circumference but not with BMI, and that lower levels of concentration and sperm count were associated with both BMI and waist circumference. This suggests that there might be some kind of a factor that is specific to abdominal fat or some other characteristic related to waist circumference (and by extension, to WHR) that influences some but not all fertility markers. On the other hand, Fejes et al. showed that sperm count, total motile sperm count, and total progressive motile sperm count were associated with both waist circumference and hip circumference, but only the first two were associated with BMI and none with WHR [24]. However, their sample was very small (*n* = 81), and therefore the conclusions drawn from that study might be somewhat limited.

One review touched upon the potential role of abdominal fat on fertility and suggested that the correlation between WHR and sex steroids may not be a direct relationship, but rather the consequence of the shared covariance of total adiposity and WHR [25]. We believe, however, that BMI and WHR as correlates for certain fertility markers (such as progressive motility in our study) might differ, and they might do so for a number of reasons. For example, in males, about 80% of biologically available estrogen is produced by the aromatization of testosterone outside the testes, primarily in the subcutaneous abdominal fat tissues [23, 26]. In addition, there might be other, thus far unknown roles of the abdominal fat in male fertility, leading to infertility in case of excess central adiposity in overweight individuals. Therefore, the importance of the distinction between BMI and WHR lies in that if there is no difference in their associations with certain fertility markers, then general weight loss might improve those markers. However, if the association is stronger for WHR than for BMI for other fertility markers, then longitudinal studies should investigate this cross-sectional finding. If longitudinal studies yield similar results, then specific strategies to reduce central adiposity might be needed in addition to general weight loss to improve those particular fertility markers – such as, potentially, progressive motility.

One limitation of our study is that we did not assess ejaculate volume. The reason for this is because we consider ejaculate volume as a secondary indicator of fertility, since the indicators regarding quality of the sperm, more specifically the concentration, progressive motility, and normal morphology are considered more important for fertility than ejaculate volume [27]. Although abstinence time in general is an influence factor for semen quality parameters, we did not add this variable to our analysis, since all patients in this study had a by the WHO prescribed optimal, and virtually the same abstinence time (3–5 days). As a matter of fact, systematic review by Hanson et al. found that semen parameters are lower before and abstinence of 3 days and higher after 5 days, but within 3 and 5 those parameters are not different [28]. Another limitation is that, since this is an exploratory study comparing BMI with WHR, we did not assess any other biological markers besides the selected fertility characteristics, or any other potential control variables besides age. Likewise, we did not control for other factors that might affect semen parameters, including but not limited to previous varicocele; hormonal, congenital, or structural abnormalities; any obvious forms of partial obstruction, or medications. However, our final dataset did not contain any patients with such conditions, and many personal background and lifestyle variables had no or just marginally significant relationship with semen parameters in prior studies [29]. Additionally, while these potential control variables may influence the relationship between sperm characteristics and either BMI or WHR, their influence will most probably be very similar on them. Moreover, numerous lifestyle factors in addition to smoking and alcohol consumption, such as use of drugs, anabolic steroids, doping, physical activity, working conditions, stress and worked hours per day might adversely affect male fertility. In our study no information about subject’s smoking or alcohol consumption were available. Although a recent review article about the relationship between infertility and lifestyle characteristics such as smoking and alcohol intake [30] found no evidence that either alcohol or smoking would influence semen quality and therefore ART therapy among infertile men, further studies should explore this. This suggests that while certain lifestyle factors might be influential among males in the general population, those might not apply to the special population of infertile men – the study population in our manuscript – whose infertility reasons might be complexly multifaceted. Given this and the fact that the aim of our analysis was to assess the potential impact of central obesity on fertility and thus the relationship between the waist-to-hip ratio and the BMI, controlling our results for these parameters would have unnecessarily complicated statistical analysis without much added benefit. Still, future studies might explore this further – by also adding other variables that have significant, and potentially confounding relationship with fertility markers – to control for any potential confounding.

## Conclusions

Our study the first to examine, using a large patient sample, the potential role of central obesity by comparing the difference between BMI and WHR as they relate to selected semen parameters. Our findings indicate a potential role of central obesity for progressive motility but not for normal morphology and concentration. Despite the limitations and the exploratory nature of this study, we can conclude that our results point to a potential role of central obesity in male infertility, but this finding should be confirmed and further explored in future research.

## Data Availability

The datasets used and/or analyzed during the current study are available from the corresponding author on reasonable request.

## Abbreviations

BMI: Body Mass Index
WHO: World Health Organization
WHR: Waist to Hip Ratio
